# Brain Resilience to Targeted Attack of Resting BOLD Networks as a Measure of Cognitive Reserve

**DOI:** 10.21203/rs.3.rs-5356022/v1

**Published:** 2024-12-04

**Authors:** Georgette Argiris, Yaakov Stern, Christian Habeck

**Affiliations:** a Cognitive Neuroscience Division, Department of Neurology, Columbia University Irving Medical Center, New York, NY; b Taub Institute, Columbia University, New York, NY

**Keywords:** cognitive aging, RANN, CR, targeted network attack, resting-state functional connectivity, longitudinal

## Abstract

Recent advancements in connectome analyses have enabled more precise measurements of brain network integrity. Identifying neural measures that can operate as mechanisms of cognitive reserve (CR) is integral for the study of individual variability in age-related cognitive changes. In the present study, we tested the hypothesis that network resilience, or the network’s ability to maintain functionality when facing internal or external perturbations that cause damage or error, can function as a CR candidate, modifying the relationship between cognitive and brain changes in a lifespan cohort of cognitively healthy adults. One hundred cognitively healthy older adults from the Reference Ability Neural Network (RANN) longitudinal lifespan cohort (50–80 years) underwent resting-state fMRI and neuropsychological testing at baseline and five-year follow-up. Using undirected weighted adjacency matrices created from the Schaefer et al. (2018) 400-parcellation atlas and 19 additional subcortical regions (419 nodes in total), whole-brain network resilience was assessed through a targeted attack approach, where nodes were sequentially removed by nodal strength and resilience defined as the iteration of the steepest slope in the largest connected component (LCC) decay. We observed that brain resilience (BR) moderated the effect of cortical thickness (CT) changes on longitudinal changes in Fluid Reasoning performance, even after adjusting for baseline differences, demographic factors, and the initial LCC of the unlesioned matrix, indicating that individuals with greater resilience were less sensitive to the effect of cortical thickness changes on changes in cognition. These findings support the use of targeted attack as a measure of cognitive reserve, suggesting that higher brain network resilience may allow individuals with reduced brain integrity to better cope with structural loss and maintain cognitive function.

## Introduction

The concept of cognitive reserve (CR) addresses individual variability in age-related cognitive outcomes, whereby some individuals withstand greater levels of brain pathology than others before manifesting cognitive decline. A recent research consortium created a consensus definition of CR as a property of the brain that allows for cognitive performance that is better than expected given the degree of life-course-related brain changes and brain injury or disease (Stern et al., 2023). This property of the brain can refer to multiple potential mechanisms, from the molecular- to network-level and should explain cognitive performance beyond the influence of brain status, the latter two ideally measured longitudinally. Quantifying at least one aspect of brain integrity is a core tenet of the CR framework to identify a genuine mechanism of CR, ensuring it is not confounded by differences in the brain’s neurobiological status that might support better cognition. The neural implementation of CR can be investigated from multiple perspectives, with prior research evidencing a task-activation-invariant CR network (Stern et al., 2018) and resting-BOLD functional connectivity pattern linked to higher IQ (Stern et al., 2021). In the current work, we explore whether a functional connectome’s resilience to network attack operates as a mechanism of CR, moderating the impact of brain change on cognitive change.

The human connectome constitutes the structural and functional network of connections within the human brain and has been described as a complex network (Sporns et al., 2005). Similar to other complex networks found in nature (e.g., social networks, the world wide web, or biological systems), the human connectome exhibits various properties that characterize complex systems, such as highly structured connectivity patterns, multiscale dynamics, small-world properties that reflect optimized information flow, and resilience to external perturbations (Sporns, 2011; Watts, 2004; Strogatz, 2001; Albert et al., 2000). Graph theory has proven to be one important framework for characterizing properties of complex networks. When applied to the topology of the brain, graphs are constructed of nodes, which represent distinct brain regions, and edges, which are their structural or functional couplings relating to information flow (Bullmore and Bassett, 2011). Then a series of network properties, or graph metrics, can be calculated to provide insight into the configuration of the network and assess strengths and weaknesses of that configuration.

With advancements in brain connectome analyses, it is possible to obtain more fine-grained measurements of brain network integrity. One measure of integrity is resilience, or the capacity of the network to retain functionality when confronted with endogenous or exogenous perturbations that result in damage or error (Gao et al., 2016). Maintenance of specific structural or functional connectivity patterns despite the deleterious effects of aging and disease is a core feature of brain resilience; in the highly resilient brain, a network should resist disconnection or disintegration in its topology even when key brain regions are attacked (Stanford et al., 2022). To obtain brain resilience estimates, virtual “lesions” are typically induced via in silico simulations, wherein nodes or edges are sequentially removed based on some pre-determined metric of significance to the network (Joyce et al., 2013; Aerts et al., 2016; Achard et al., 2006).

Computational modeling of lesion effects in the human brain has drawn a link between localized structural damage of networks and global network disruptions that could impact behavioral and cognitive outcomes (Alstott et al., 2009). Preservation of specific functional network metrics, such as global system segregation and modularity (Ewers et al., 2021; Varangis et al., 2020), local efficiency (Adams et al., 2023), and robustness of core network topology (Stanford et al., 2022) has been linked to cognitive resilience in both healthy aging and Alzheimer’s disease. Additionally, prior work has displayed a link between network integration measured across graph filtration thresholds and fluid reasoning performance in a longitudinal aging cohort (Argiris et al., 2023). Investigating dynamic changes of network topology through iterative processes, emphasizing metrics related to nodal importance, could provide valuable insights into the mechanisms underlying cognitive changes. Moreover, the brain’s capacity to retain network functionality and resist perturbations may serve as a potential mechanism of CR, wherein individuals with higher brain resilience may better resist the pathological impacts of brain “hardware” malfunctions and sustain higher level of cognitive functioning (Stern et al., 2023; Stern et al., 2009).

Here, we investigated whether a measure of brain resilience, based on in silico lesioning of the largest connected component (LCC) of the network, can possibly serve as a mechanism of CR in healthy older adults. Recent prior work has demonstrated the LCC as a measure of structural network integrity (Joyce et al., 2013; Alstott et al., 2009; Watts & Strogatz, 1998). Additionally, Menardi and colleagues (2021) analyzed brain resilience of LCC to targeted attack in twins and found moderate heritability several topological network measures, including the critical point in the drop of the LCC. Additionally, we wanted to establish to what extent the critical point of drop over lesioning iterations can offer predictive utility in explaining cognitive change beyond the information encoded in the static connectome.

We utilized longitudinal resting-state and neuropsychological data from the Reference Ability Neural Network (RANN) and Cognitive Reserve (CR) cohorts to test for moderation between changes in structural brain integrity and brain resilience as it relates to out-of-scanner task performance. We used cortical thickness as our longitudinal measure of brain integrity. This measure has been shown to decline throughout the lifespan (Fjell et al., 2015) and is a predictor of cognitive performance (Dominguez et al., 2021). Additionally, cross-sectional research from our lab has demonstrated negative associations between age and cortical thickness (CT), while also uncovering a complex relationship between CT and other demographic factors (Habeck et al., 2020). Finally, in order to balance network properties such as efficiency and robustness, brain networks exhibit distinctive configurations that enable efficient local processing and global integration of their components (Rubinov and Sporns, 2010; Deco et al., 2015) to ensure high levels of resilience (Achard et al., 2006; Joyce et al., 2013). As small-world organization is thought to reflect this balance (Watts, 2004; Albert et al., 2000), we also tested its relationship to the LCC and behavior. Ultimately, we hypothesized that individuals with higher brain resilience will sustain larger LCCs over longer iterations of in silico lesioning before decay in LCC (i.e., critical drop) becomes evident and that more resilient individuals will be less susceptible to the effects of age-related brain changes on behavioral performance.

## Methods

### 2.1. Participants

Analyses included one hundred native English speaking, right-handed (Oldfield Edinburgh Handedness Inventory; Oldfield, 1971) older adults (age= 64.22 ± 7.33; range= 50 – 80 years) from the Reference Ability Neural Network (RANN) or the Cognitive Reserve (CR) cohort, both community-based cohort from the greater New York area. Both cohorts employed identical inclusion/exclusion criteria, structural and resting state functional imaging protocols, as well as a considerable overlap in cognitive assessments and questionnaires. The main contrast between the two studies lies in the functional task-based imaging protocols, which won’t be elaborated on here. Participants were tested at two time points—baseline and 5-year follow-up. Participants were screened for psychiatric and medical conditions, uncorrectable hearing and vision loss, and any other contraindications that could have impeded MRI acquisition prior to study participation. Additionally, participants were tested at both time points for dementia and mild cognitive impairment using the Dementia Rating Scale (DRS; Mattis, 1988). All participants had less than 30% of their functional data removed and interpolated (i.e., scrubbed) due to motion artifact (Parkes et al., 2018; Power et al., 2012). See Table 1 for a list of participant demographics.

### 2.2. Procedure

Participants were scanned during a period of rest prior to the task-based imaging protocol during which cognitive tasks were performed in-scanner. Only resting state data will be considered here. Participants additionally underwent neuropsychological assessment in a prior session. The relationship between out-of-scanner neuropsychological performance and resting-state connectivity will be analyzed.

#### 2.2.1. Neuropsychological test assessment

All participants underwent an out-of-scanner comprehensive neuropsychological battery to assess an array of cognitive functions. The tasks were administered in the following fixed sequence: Wechsler Adult Intelligence Scale (WAIS-III; Wechsler, 1997), Letter-Number Sequencing, American National Adult Reading Test (AMNART; Wechsler, 1997), Selective Reminding Task (SRT) immediate recall (Buschke and Fuld, 1974), WAIS-III Matrix Reasoning (Wechsler, 1997), SRT delayed recall and delayed recognition (Buschke and Fuld, 1974), WAIS-III Digit Symbol (Wechsler, 1997), Trail-Making Test versions A and B (TMT-A/B; Reitan, 1978), Controlled Word Association (C-F-L) and Category Fluency (animals; Benton et al., 1983), Stroop Color Word Test (Golden, 1975), Wechsler Test of Adult Reading (WTAR; Holdnack, 2001), WAIS-III Vocabulary (Wechsler, 1997), and WAIS-III Block Design (Wechsler, 1997).

Previous analyses demonstrated that the administered tasks represent distinct latent variables for four cognitive domains (Razlighi et al., 2016; Salthouse & Ferrer-Caja, 2003). The four cognitive domains represented were: Episodic Memory (three measures from the SRT); Fluid Reasoning (WAIS Matrix Reasoning, WAIS Block Design, and TMT-B); Perceptual Speed (WAIS Digit Symbol, 2 measures from the Stroop test, TMT-A); Vocabulary (WAIS Vocabulary, AMNART, WTAR). In this case, the three tasks were averaged within each domain.

To standardize comparisons between tasks, behavioral scores at each time point were z-transformed using the mean and standard deviation calculated across all participants for each task separately at baseline. Since speed tasks were measured as reaction time, z-scores were inverted so that higher scores always indicate better performance.

#### 2.2.2. fMRI Data Acquisition

##### 2.2.2.1. Scan Parameters

Image acquisition was performed using a 3T Philips Achieva Magnet. The resting state protocol consisted of fMRI scans collected for a period of either 5 minutes (baseline: *n* = 46; follow-up: *n* = 0) or 9.5 minutes (baseline: *n* = 54; follow-up: *n* = 100) minutes. A T1-weighted image of the whole brain was performed for each subject with a Magnetization Prepared Rapid Gradient Echo (MPRAGE) sequence with the following parameters: TE/TR of 3/6.5 ms, flip angle of 8°, in-plane resolution of 256 × 256 voxels, field of view (FOV) of 25.4 × 25.4 cm, and 165–180 slices in the axial direction with a slice-thickness/gap of 1/0 mm. All scans used a 240 mm field of view. For the fMRI blood oxygen level-dependent (BOLD) resting state scans, the following parameters were used: TE/TR of 20/2000 ms, flip angle of 72°, in-plane resolution of 112 × 112 voxels, and a slice thickness/gap of 3/0 mm and 37 slices. A neuroradiologist analyzed each participant’s scan for any anomalies, and noteworthy observations were subsequently conveyed to the participant’s primary healthcare provider.

##### 2.2.2.2. Data Processing

Images were preprocessed using an in-house developed native space method (Razlighi et al., 2014). In brief, the preprocessing pipeline was as follows: slice timing and motion correction (MCFLIRT) were applied using the FSL package (Jenkinson et al., 2012). Registration to the middle volume was performed with 6 degrees of freedom, 256 bins mutual information, and sinc interpolation. Frame-wise displacement (FWD) and root-mean-square difference (RMSD) were computed from motion parameters and BOLD percentage signal, with a conservative RMSD threshold of 0.3%. Detected contaminated volumes, meeting criteria of FWD > 0.5 mm or RMSD > 0.3%, were replaced by linearly interpolated adjacent volumes prior to temporal filtering (Carp, 2013). Subsequently, motion-corrected signals underwent bandpass filtering (0.01 to 0.09 Hz) using Flsmaths–bptf. Finally, data were residualized by regressing out FWD, RMSD, white matter signals, and lateral ventricular signals (Birn et al., 2006); finally, each T1 image was aligned to the 2mm MNI template using advanced normalization tools (ANTs).

#### 2.2.3. Functional Connectivity

**Figure 1.**
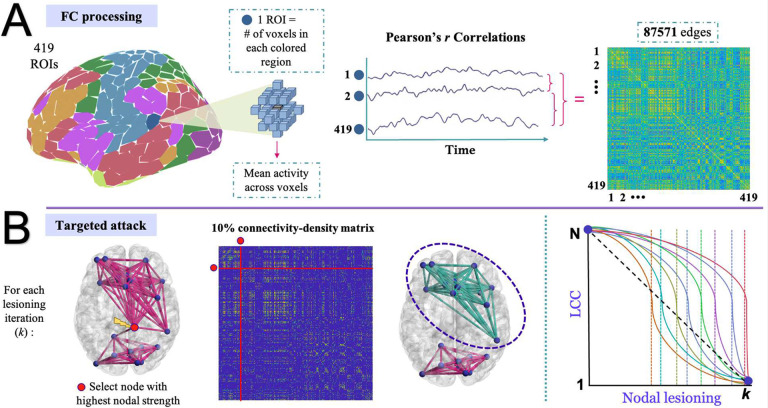
Schematic of functional connectivity (FC) matrix generation and application of targeted attack. Panel A: Time-series extraction for each region of Schaefer’s 400 parcellation scheme in addition to the 19 subcortical regions provided by the human connectome project. Time-series data were averaged across all voxels within each region, and a cross-correlation connectivity matrix using Pearson’s *r* was created from all nodal pairings. Brain parcellation image was created with the ggsegSchaefer library in R (Mowinckel & Vidal-Piñeiro D, 2022). Panel B: Each participant’s connectivity matrix was thresholded at a 10% connection density to retain the strongest (positive) connections. In the toy brain example, the complete network (left) represents the largest connected component (LCC). The node with the highest nodal strength is identified and removed (red), and the resulting cluster (green) represents the new LCC. The plot on the far right shows potential curves across nodal lesioning iterations (k = N, where N is the maximum number of nodes, 419). Each colored curve represents an individual, with colored dashed lines indicating possible critical drop-off points or maximum deflections, where the number of lesioning iterations sustained distinguishes between a low resilient individual (i.e., orange curve/dashed line) versus a high resilient individual (red curve/dashed line). The dashed diagonal black line merely indicates hypothetical LCC drop under random attack, which was not focused here.

T1 image segmentation was performed using FreeSurfer (Dale et al., 1999). We utilized the Schaefer atlas with 400 parcels, which divides the cortex into 400 distinct regions based on functional and anatomical features. Several parcellation schemes exist for the Schaefer atlas, ranging from 100 to 1000 parcels and divided into either 7 or 17 functional networks. Given that there is an estimated 300–400 defined human neocortical areas (Van Essen et al., 2012), we opted for the 400-ROI parcellation scheme for greater precision, as it has also been suggested that lower-resolution parcellations may not be able to fully differentiate cortical areas (see Schaefer et al., 2018; Gordon et al., 2016). Additionally, 19 subcortical grey matter structures were added from a parcellation map provided by the Human Connectome Project (). Thus, a total of 419 regions-of-interest (ROIs) were considered. To obtain regional time series, the application of the 419 ROI parcellation scheme was performed in native space, where ROIs (i.e., nodes) were transferred to each participant’s T1 space via non-linear registration of an individuals’ structural scan to the MNI template using the ANTS software package. Each node was intersected with a patient’s dilated gray matter mask in MNI space, which is a slight expansion around the gray matter boundary to account for small inaccuracies during the segmentation or registration process. An intermodal, intra-subject, rigid-body registration of the fMRI reference image and T1 scan was performed with FLIRT with 6 degrees of freedom, normalized mutual information as the cost function (Jenkinson and Smith, 2001), and then used to transfer the 419 nodal masks from T1 space to fMRI space. All the voxels within each nodal mask were averaged to obtain a single fMRI time-series per node. Pearson correlations were then performed for all pairwise nodal combinations, which resulted in 419 × (418/2) = 87,571 fMRI connectivity pairs (see Figure 1A for FC schematic). Cross-correlation matrices of time-series were derived without regressing out the global signal average, as this procedure can alter the correlation pattern and magnitude (Alstott et al., 2009).

#### 2.2.4. Targeted Attack

Prior to targeted attack, participant matrices underwent density-based thresholding to retain only the strongest connections. Previous work has shown measures of network topology to be sensitive to different types of thresholding (van Wijk et al., 2010) in addition to age-related effects emerging only in certain network metrics and range of connectivity thresholds (Varangis et al., 2021; Geerligs et al., 2015; Iordan et al., 2017). In the current analysis, we adopted a primary connectivity-density threshold of 10% for several reasons (1) this threshold falls within the range used in previous studies; (2) it was employed in the targeted attack study by Menardi and colleagues (2021); and (3) a 10% sparsity threshold has been associated with higher test-retest reproducibility for global network metrics (Wang et al., 2011). However, as a sensitivity analysis, we also repeated the analysis using two additional thresholds, 5% and 15%, around the 10% threshold. A 10% density threshold was applied to each participant’s individual connectivity matrix (nodes surviving threshold— baseline: *M=*415.21, *SD*=5.72; follow-up: *M=*414.45, *SD*=8.97).

For each individual’s connectivity matrix, four graph theory metrics were extracted using Brain Connectivity Toolbox (Rubinov et al., 2009) functions implemented in MATLAB:

1. *clustering coefficient*: a measure of network segregation, defined as the ratio between the number of connections that exist between direct neighbors of a node and the maximum number of possible connections, averaged across all network nodes.
2. *characteristic path length*: a measure of integration, defined as the average shortest path length (i.e., minimum number of connections to link two nodes) across all network pairs
3. *global efficiency*: defined as the average inverse of the shortest path length between all nodal pairs.
4. *largest connected component (LCC)*: defined as the component of the graph that contains the largest number of nodes that are connected by edges.

For each participant’s original connectivity matrix, we also generated random matrices that preserved the number of nodes and degree distribution (Maslov & Sneppen 2002). To ensure sufficient randomization, each edge was rewired over 100 iterations. These randomized matrices served as null networks, enabling the normalization of the graph theory metrics derived from the original matrices. The clustering coefficient and characteristic path length of the original matrices, normalized by the values derived from random matrices, were used to calculate the small-world network property of each participant’s graph *prior* to lesioning. It was computed as follows:

1. Let *CPL*[*p*] the characteristic path length, and *CC*[*p*] the clustering coefficient for each participant *p*. Let *CC*_rand_ represent the clustering coefficient and *CPL*_rand_ the characteristic path length of the randomized matrix.
2. Define *SW*[*p*] as the small-world network expressed as:
$$\text{SW}[p]=\frac{\text{CC}([p])/{\text{CC}}_{\text{rand}}[p]}{\text{CPL}([p])/{\text{CPL}}_{\text{rand}}[p]}$$

Brain resilience was computed via the sequential removal of nodes from the weighted adjacency matrix, based on prior lesioning studies (e.g., Albert et al., 2000; Albert and Barabási, 2002; Alstott et al., 2009; Menardi et al., 2021). Nodes were sorted in descending order based on their nodal strength. We chose nodal strength as the organizing principle for targeted attack in order to preserve more information by summing the weights across all edges, rather than simply binarizing to obtain the cardinal quantity of connections. However, Alstott and colleagues (2009) demonstrated that targeted attack by either degree or strength produce almost identical results. At each iteration of attack, nodal strength was calculated to account for the effect of prior lesioning and the node with the highest nodal strength, along with all of its connections, was removed from the graph; graph theory metrics were then extracted. The sequence of steps can be expressed as follows:

1. Let *A* be the *N* × *N* connectivity matrix where *A*_*ij*_ represents the Pearson’s *r* connectivity value between node *i* and node *j*.
2. Calculate each graph theory metric on the initial connectivity matrix where A^(0)^=*A*.
3. For each iteration *k* from 1 to *N*:
  - Calculate the nodal strengths for each node *i* in the matrix ^(*k*–1)^:
$$s_{i}^{\left(k-1\right)}=\sum_{j\;\in \;I_{k-1}}A_{\text{ij}}^{\left(k-1\right)}$$
    where *I*_*k*−1_ is the set of indices of the remaining nodes in *A*^(*k*–1)^.
  - Find the node *A*_*k*_ with the highest nodal strength:
$$n_{k}=\underset{j\;\in \;I_{k-1}}{\text{arg}\;\text{max}\;}s_{i}^{\left(k-1\right)}$$
  - Update the set of remaining nodes with the removal of *A*_*k*_:
$$I_{k}=I_{k-1}\;\backslash \;\left\{n_{k}\right\}$$
  - Update the connectivity matrix by removing the rows and columns corresponding to *A*_*k*_ :
$$A^{\left(k\right)}=A^{\left(k-1\right)}\left(I_{k},I_{k}\right)$$
  - Calculate each graph theory metric on the updated matrix.

Our primary measure of brain resilience was based on the rate of change in the LCC of the graph. In a fully connected graph, the LCC initially includes all the nodes; however, as nodes are iteratively removed, the graph becomes increasingly fragmented until the LCC is reduced to a single node (the last remaining node; see Figure 1B). We inferred that more resilient individuals would sustain larger LCCs over longer iterations of lesioning before decay in LCC becomes evident (i.e., critical point), with resilience operationalized as the iteration of steepest slope drop in LCC. The LCC under targeted attack was not normalized to the LCC of a random graph because the primary goal was to measure the direct impact of targeted attack on the network; absolute resilience of the network is measured without introducing unnecessary complexity.

The critical point was calculated as follows:

1. Let *lcc*[*p*, *i*] be the value of the LCC for participant *p* at iteration *i*.
2. Define *LCC*_slope_[*p*, *i*] as the slope between points *i* and *i*+1 for participant *p*.
3. Calculate the slope for each participant *p* and each point *i*:
$${\text{LCC}}_{\text{slope}}[p,\;i]=\text{lcc}[p,\;i+1]-\text{lcc}[p,\;i]$$
4. Define *LCC*_drop_ as the minimum slope for each participant *p* across the *n* node set:
$${\text{LCC}}_{\text{drop}}[p]=\underset{i}{\text{min}}\left({\text{LCC}}_{\text{slope}}[p,\;i]\right)\;\text{for}\;i\in \{1,2,\ldots ,n-1\}$$

#### 2.2.5. Analytic approach

Linear regression analyses with change score models were utilized. Demographic variables including age, NART IQ (NART), education (Edu), and sex were included in all regression models. Cortical thickness was also considered as variable of interest. Scrubbing percentage was included as a nuisance variable in all models. Model results are reported at a p < 0.05 uncorrected threshold. To standardize comparisons between tasks, behavioral scores at each time point were z-transformed using the mean and standard deviation calculated across all participants for each task separately at baseline. Since speed tasks were measured as reaction time, z-scores were inverted so that higher scores always indicate better performance.

For regressions of longitudinal change, change values were calculated as follow-up (FU) minus baseline (BL). For change in cortical thickness (ΔCT), the change values were residualized with respect to baseline measurements. For behavioral performance, change values were not residualized with respect to baseline, as we were interested in explicitly modeling baseline effects.

## Results

**Figure 2.**
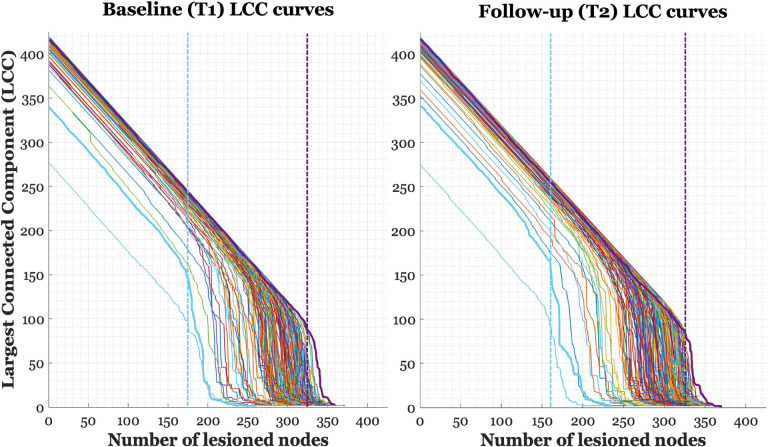
Line plots of each participant’s largest connected component (LCC) curve at each time point, when iteratively removing nodes by nodal strength. Vertical dashed lines indicate the largest slope of decay for two example participants, where the early slope drop (blue) suggests a less resilient individual than one with late slope drop (purple).

The main results are reported for the primary connectivity-density threshold of 10%. In anticipation of our findings, results for models using the 5% and 15% connectivity-density thresholds are reported in Supplementary Data Table 1 (ST1) for the FLUID domain. Additionally, as the scan length differed between participants and time point (i.e., 5 or 9.5 minutes), we also performed a sub-analysis on the truncated time series, with results again reported in ST1. For a visualization of the LCC lesioning curves at both baseline and follow-up, see Figure 2. We tested brain resilience as a measure of CR by utilizing the *LCC*_drop_ at follow-up, or time 2 (${\text{LCC}}_{{\text{drop}}^{\text{T}2}}$) and critically focusing on the interaction between the ${\text{LCC}}_{{\text{drop}}^{\text{T}2}}$ and ΔCT in the behavioral models. The ${\text{LCC}}_{{\text{drop}}^{\text{T}2}}$ was first standardized to reduce multicollinearity between the two predictors and for comparability across models. Additionally, some evidence suggests that healthy structural and functional brain networks exhibit small-world topology (Stam, 2010), this property being integral to the study of network robustness (Albert et al., 2000; see Aerts et al., 2016), we investigated the relationship between small-world architecture of the original unlesioned graphs and both network resilience and behavioral performance.

### 3.1. Behavioral models

**Figure 3.**
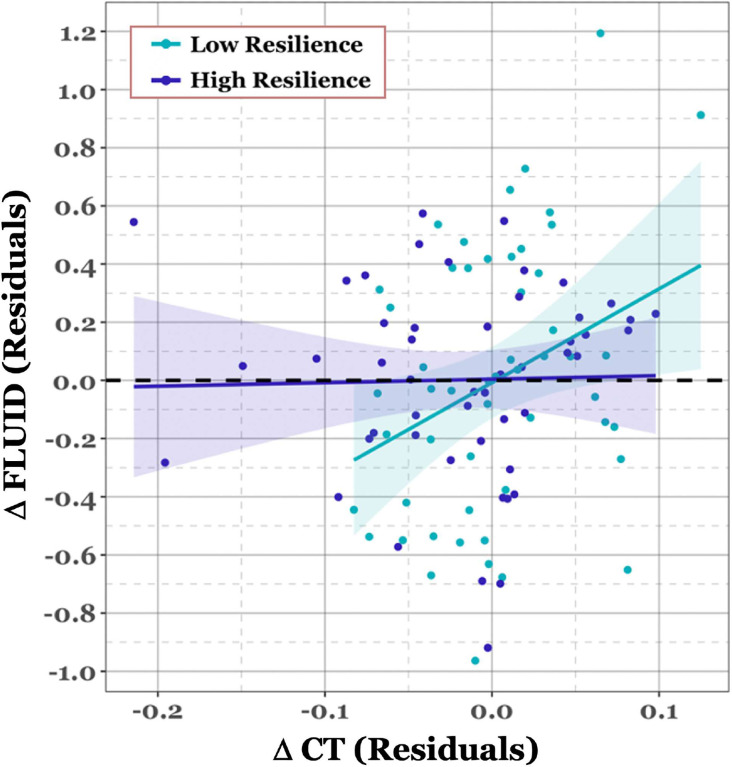
Scatterplot of the relationship between longitudinal change in Fluid Reasoning performance and cortical thickness, divided by brain resilience (i.e., ${\text{LCC}}_{{\text{drop}}^{\text{T}2}}$). The change in fluid reasoning performance (Δ FLUID) represents the residuals after adjusting the model for all factors, excluding ΔCT and ${\text{LCC}}_{{\text{drop}}^{\text{T}2}}$. ΔCT represents the residuals after adjusting change values (T2 – T1) for baseline CT. Resilience was determined by median split in standardized scores in ${\text{LCC}}_{{\text{drop}}^{\text{T}2}}$.

We investigated the effect of demographic factors, brain integrity (ΔCT), and brain resilience (${\text{LCC}}_{{\text{drop}}^{\text{T}2}}$) on change in behavioral performance for each cognitive domain. We also controlled for scrubbing percentage at time 2 (Scrub%_T2_), initial LCC at time 2 (${\text{LCC}}_{\text{k}=0^{\text{T2}}}$), and baseline behavioral performance. For a full list of predictors and parameters, see table 2. For all cognitive domains, baseline performance significantly predicted the change in performance over time. For MEM, FLUID, and SPEED, this relationship was negative, such that higher baseline performance predicted less change over time. For VOCAB, this relationship was positive; however, there was also a significant negative effect of NART, such that higher NART predicted less change over time. Additionally, as can be observed from the model estimates, the standardized beta coefficients indicated potential multicollinearity, which was confirmed for the variables NART and baseline performance by variance inflation factor. Age was also significantly linked to longitudinal behavioral declines for MEM, FLUID, and SPEED, but not for the VOCAB domain. The most notable findings overall were for the FLUID domain, where both ΔCT and the interaction between ΔCT and the ${\text{LCC}}_{{\text{drop}}^{\text{T}2}}$ both significantly predicted change in fluid reasoning over time. ΔCT was positively linked to behavioral change as expected, where higher brain integrity over time was related to better behavioral performance. Critically, there was a negative interaction between ΔCT and the ${\text{LCC}}_{{\text{drop}}^{\text{T}2}}$, where individuals with higher brain resilience were less sensitive to the effects of change in structural integrity on behavioral performance (see Figure 3). This interaction between ΔCT and the ${\text{LCC}}_{{\text{drop}}^{\text{T}2}}$ held up when we considered density thresholds of both 5% and 15% in addition to the shorter truncated time series at 10% density threshold (see Supplementary Table 1).

### 3.2. Relationship between small-world and brain resilience

We investigated the relationship between static small-world property of each individual’s unlesioned matrix at follow-up (i.e., *SW*_T2_) and (${\text{LCC}}_{{\text{drop}}^{\text{T}2}}$). We found that both the initial LCC on the unlesioned matrix ${\text{LCC}}_{\text{k}=0^{\text{T2}}}$ and *SW*_T2_ both significantly positively predicted the ${\text{LCC}}_{{\text{drop}}^{\text{T}2}}$; that is, higher initial LCC and small-world property of the network was linked to higher resilience (see Table 3). When we added *SW*_T2_ to the FLUID model to see if it accounted for any variance in ΔFLUID, the interaction between ΔCT and the ${\text{LCC}}_{{\text{drop}}^{\text{T}2}}$ remained significant (β = −0.217, 95% CI [−.126 −.009], *p* = 0.024, 𝜂_*ρ*_^2^ = 0.057).

Accounting for baseline differences and covariates, we found a significant negative interaction between brain resilience and Δ CT on Δ Fluid Reasoning performance. Our finding supports evidence for targeted attack as a measure of CR, where higher brain network resilience may have permitted individuals with reduced structural brain integrity to better cope with structural loss by enhance preservation of cognitive function.

## Discussion

In the present paper, our main objective was to investigate whether greater brain resilience, as assessed via targeted attack of resting-state functional connectivity networks, acted as a mechanism of CR among older age adults. We utilized the LCC of the network as our topological feature of interest, identifying the critical point of LCC drop, which served as our measure of resilience; this measure has frequently been employed in the literature when quantifying the robustness of a network under targeted, sustained attack (Albert et al., 2000; Albert et al., 2002; Joyce et al., 2013; Menardi et al., 2021). Critically, we observed a moderation effect of this critical point of LCC-drop on the relationship between structural brain changes and cognitive performance changes notably for the fluid reasoning domain. To our knowledge, this is the first study to identify a metric of targeted attack that functioned as a mechanism of CR, in line with the recent framework established for the study of resilience and reserve (Stern et al., 2023).

Prior work in simulated network attack has typically aimed to compare network robustness under different attack strategies to infer to what extent a biological network displays specific architectures such as scale-free, small-world, and random networks (Albert et al., 2002; Albert et al., 2000; Achard et al., 2006). Targeted attacks are often compared to random attacks to evaluate the robustness and vulnerability of the network. A random attack involves the arbitrary removal of nodes or edges, representing random failures or disruptions. In contrast, targeted attack specifically removes the most critical nodes, such as those with the highest degree, strength, or centrality, simulating a strategic assault on the network’s most influential components. Comparing these different attacks offers insight into underlying network structure and resilience patterns; whereas small-world topology, for instance, is typically robust to random attacks, given its distributed and highly clustered configuration, it is more vulnerable to targeted attack of its hubs. In the present work, our aim was not to verify or refute network structure by comparing targeted versus random attack, but to directly compare individual differences in brain resilience to targeted attack and elucidate its relationship to structural and cognitive changes. However, given the evidence for small-world topology of brain networks (see Bassett & Bullmore, 2017; Watts & Strogatz, 1998), we also investigated the relationship between the LCC drop and small-world property of the unlesioned connectivity matrix. Regression analysis revealed that both the initial LCC and the small-world properties of the unlesioned matrix independently and positively predicted the LCC drop. Prior work has indeed shown that this property is integral to the study of network robustness (Albert et al., 2000; see Aerts et al., 2016); small-world architecture not only enhances synchronization and information flow but also supports diverse neural computations by integrating local processing across distributed networks (Bassett & Bullmore, 2017).

The fact that brain resilience moderated the relationship between structural brain changes and cognitive changes, specifically in the Fluid Reasoning domain, is particularly noteworthy. Prior work in the lab examined changes in the number of graph components across various connectivity filtration thresholds as an indicator of network integrity. The findings showed that a longer transition from isolated nodes to a single connected component was positively associated with better longitudinal performance in fluid reasoning over time (Argiris et al., 2023). Interestingly, this finding was observed in task-based functional connectivity; here, we find a relationship between higher brain resilience during resting-state connectivity and out-of-scanner neuropsychological task performance. Some work has supported the idea that task-based functional connectivity better predicts both cognitive task activations (Cole et al., 2021) and as well as cognitive performance when studied in the context of a global neural flexibility (Varangis et al., 2022). It could be that the relationship between resting-state functional connectivity and cognition may depend on both the network property and/or the cognitive domain being measured. Notably, higher fluid intelligence has been linked to more efficient brain connectivity updates in network reconfiguration, indicating smaller changes in functional network architecture between task and rest (Schultz & Cole, 2016). It is important to highlight the distributed nature of networks underlying fluid reasoning processing (Colom et al., 2010). Given the robust association between fluid reasoning and whole-brain network metrics, it may be particularly insightful to focus on fluid reasoning when studying both healthy and potentially pathological aging.

One important point of consideration is the fact that our brain resilience measure was calculated on weighted graphs that were thresholded to retain the strongest connections. Despite this being a common practice in the literature (Sporns & Bullmore, 2009; Van Wijk et al., 2010), more recent work has argued for the importance of considering weak connections in neural systems (Bassett & Bullmore, 2017). Importantly, some work has shown that individual differences in the strength of weak functional connections from the prefrontal cortex to the frontoparietal network predict differences in fluid intelligence (Cole et al., 2012). Our decision to threshold the weighted matrices was due to the limited variability in our network measure of consideration (i.e., LCC) when no threshold was applied. To better capture individual differences in brain resilience while still maintaining robust analytical validity, we evaluated the consistency of our findings across different threshold levels; our findings upheld across all thresholds. Additionally, we repeated the analysis with the truncated time series, again replicating our original findings; this is critical, despite work showing that greater reliability is found at longer scan lengths (Birn et al., 2013). In the present work, we also only considered the point of drop in the LCC, via successive nodal removal, as our measure of brain resilience. Other work has focused not only on other nuanced elements of the lesioning curve, but also on targeted edge removal as a potentially more sensitive measure of resilience (Menardi et al., 2021). Finally, a logical next step should involve real-world application to analyze dynamic network reconfiguration under attack. Transcranial magnetic stimulation can induce theory-guided perturbations to validate or challenge in silico predictions of sites critical for network integrity.

## Supplementary Material

1

## Figures and Tables

**Table 1.** Participant demographics divided by decade of life. Mean (M) and standard deviation (SD) are presented for age, NART, and Education.

Age Bracket	N	Sex		Age		NART		Education	
		Male	Female	M	SD	M	SD	M	SD
50–59 years	24	10	14	54.08	3.05	119.49	7.54	15.79	2.15
60–69 years	52	29	23	64.77	2.78	119.34	7.69	16.02	2.36
70–80 years	24	14	10	73.46	2.70	119.23	8.35	16	2.36

**Table 2.** List of predictors for all linear regression behavioral models. Significant predictors are highlighted in purple. Change (Δ) in behavior of each domain (MEM= memory; FLUID= fluid reasoning; SPEED= processing speed; VOCAB= vocabulary) is the outcome variable. We also controlled for scrubbing (Scrub%_T2_), initial (LCC ${\text{LCC}}_{\text{k}=0^{\text{T2}}}$), and baseline behavioral performance in each model. Change in cortical thickness (ΔCT: T2 – T1) was residualized with respect to baseline. NOTE: The standardized beta coefficient exceeds one for the relationship between NART and ΔVOCAB due to high multicollinearity between VOCAB_T1_ and NART; model effects should be cautiously interpreted.

Outcome	Predictor	β	p	CI	𝜂_ρ_^2^
	Age	−.341	<001***	[−.052 −.014]	.114
	Edu	.162	.184	[−.024 .125]	.020
ΔMEM	Sex	.080	.408	[−.157 .382]	.008
	NART	−.053	.672	[−.028 .018]	.002
	MEM_t1_	−.582	<001***	[−.551 −.192]	.160
	Scrub%_T2_	.098	.968	[−.020 .021]	.000
	${\text{LCC}}_{{\text{drop}}^{\text{T}2}}$	−.101	.197	[−.407 .085]	.010
	${\text{LCC}}_{\text{k}=0^{\text{T2}}}$	.133	.127	[−.005 .042]	.026
	ΔCT	.208	.837	[−2.358 2.904]	.001
	${\text{LCC}}_{{\text{drop}}^{\text{T}2}}*\Delta \text{CT}$	−.218	.076	[−5.076 .261]	.035
	Age	−.250	.011*	[−.027 −.004]	.071
	Edu	−.009	.939	[−.047 .044]	.000
ΔFLUID	Sex	.098	.284	[−.075 .254]	.013
	NART	.303	.025*	[.003 .036]	.056
	FLUID_t1_	−.582	<001***	[−.465 −.202]	.223
	Scrub%_T2_	.098	.337	[−.006 .018]	.011
	${\text{LCC}}_{{\text{drop}}^{\text{T}2}}$	−.101	.553	[−.200 .108]	.000
	${\text{LCC}}_{\text{k}=0^{\text{T2}}}$	.133	.429	[−.009 .021]	.007
	ΔCT	.208	.036*	[.109 3.278]	.029
	${\text{LCC}}_{{\text{drop}}^{\text{T}2}}*\Delta \text{CT}$	−.218	.023*	[−3.517 −.264]	.057
	Age	−.343	.002**	[−.037 −.009]	.107
	Edu	.171	.180	[−.017 .091]	.020
ΔSPEED	Sex	.123	.224	[−.074 .314]	.017
	NART	.084	.525	[−.012 .022]	.005
	SPEED_t1_	−.233	.043*	[−.308 −.005]	.045
	Scrub%_T2_	.202	.054	[−.001 .029]	.041
	${\text{LCC}}_{{\text{drop}}^{\text{T}2}}$	−.143	.451	[−.255 .114]	.008
	${\text{LCC}}_{\text{k}=0^{\text{T2}}}$	.190	.302	[−.008 .027]	.012
	ΔCT	−.088	.419	[−2.659 1.116]	.006
	${\text{LCC}}_{{\text{drop}}^{\text{T}2}}*\Delta \text{CT}$	.037	.727	[−1.614 2.305]	.001
	Age	−.069	.506	[−.012 .006]	.005
	Edu	.082	.539	[−.026 .050]	.004
ΔVOCAB	Sex	−.039	.695	[−.156 .105]	.002
	NART	−1.205	.002**	[−.085 −.020]	.107
	VOCAB_t1_	.899	.013*	[.082 .661]	.069
	Scrub%_T2_	.020	.846	[−.009 .011]	.000
	${\text{LCC}}_{{\text{drop}}^{\text{T}2}}$	−.207	.269	[−.190 .054]	.021
	${\text{LCC}}_{\text{k}=0^{\text{T2}}}$	.211	.252	[−.005 .018]	.015
	ΔCT	−.117	.291	[−1.970 .598]	.004
	${\text{LCC}}_{{\text{drop}}^{\text{T}2}}*\Delta \text{CT}$	.186	.092	[−.192 2.517]	.032

Asterisks indicate statistical significance at threshold levels p<0.05 (*), p<0.01 (**), and p<0.001(***).

*β*= Standardized coefficient beta; *p*= p-value (uncorrected); *CI*= 95% confidence interval; 𝜂_*ρ*_^2^ = partial eta-squared effect size.

**Table 3.** List of predictors for the model with ${\text{LCC}}_{{\text{drop}}^{\text{T}2}}$ *as outcome*. Significant predictors are highlighted in purple. We also controlled for scrubbing (Scrub%_T2_) and initial LCC (${\text{LCC}}_{\text{k}=0^{\text{T2}}}$).

Outcome	Predictor	β	p	CI	𝜂_ρ_^2^
	Age	−.027	.474	[−.0396 .184]	.006
	Edu	−.003	.939	[−1.115 1.032]	.0001
${\text{LCC}}_{{\text{drop}}^{\text{T}2}}$	Sex	.023	.492	[−2.457 5.07]	.005
	NART	.005	.927	[−.308 .337]	.0001
	${\text{LCC}}_{{\text{drop}}^{\text{T}2}}$	.033	.371	[−.045 .119]	.009
	Scrub%_T2_	−.007	.847	[−.309 .254]	.0004
	${\text{LCC}}_{\text{k}=0^{\text{T2}}}$	.461	<001***	[1.022 1.511]	.541
	CT_T2_	−.005	.892	[−21.904 19.102]	.0002
	*SW*_T2_	.575	<001***	[25.915 34.77]	.673

Asterisks indicate statistical significance at threshold levels p<0.05 (*), p<0.01 (**), and p<0.001(***).

*β*= Standardized coefficient beta; *p*= p-value (uncorrected); *CI*= 95% confidence interval; 𝜂_*ρ*_^2^ = partial eta-squared effect size.

## Data Availability

The data that support the findings of this study are available from the corresponding author upon reasonable request. Custom-written code detailing analysis can also be made available upon reasonable request.
