# Supplementary material for: Brain Resilience to Targeted Attack of Resting BOLD Networks as a Measure of Cognitive Reserve
Source: Res Sq. 2024 Dec 4:rs.3.rs-5356022. Preprint. [Version 1] doi: 10.21203/rs.3.rs-5356022/v1 (PMC11643323; doi:10.21203/rs.3.rs-5356022/v1)
Supplement: 1 [file NIHPPrs5356022v1-supplement-1.pdf]

Supplemental Material

	Predictor	***β***	***p***	***CI***	η_ρ^2
**5% Density Threshold**	Age	-.242	.015*	[-.027 -.003]	.066
	Edu	-.018	.878	[-.049 .042]	.0003
	Sex	.089	.335	[-.085 .246]	.011
	NART	.328	.016*	[.004 .038]	.064
	$FLUID_{T1}$	-.612	<.001***	[-.483 -.217]	.237
	$Scrub\%_{T2}$	.085	.382	[-.007 .018]	.009
	$LCC_{drop^{T2}}$	-.180	.316	[-.245 .080]	.007
	$LCC_{k=0^{T2}}$	.176	.327	[-.003 .009]	.011
	ΔCT	.186	.055	[-.031 3.053]	.031
	$LCC_{drop^{T2}}$ * ΔCT	-.187	.046*	[-3.329 -.035]	.045
**15% Density Threshold**	Age	-.237	.015*	[-.027 -.003]	.065
	Edu	-.027	.811	[-.051 .040]	.001
	Sex	.115	.214	[-.062 .271]	.017
	NART	.329	.015*	[.004 .038]	.065
	$FLUID_{T1}$	-.580	<.001***	[-.464 -.200]	.220
	$Scrub\%_{T2}$	.105	.275	[-.006 .019]	.014
	$LCC_{drop^{T2}}$	-.120	.363	[-.174 .064]	.001
	$LCC_{k=0^{T2}}$	.183	.162	[-.006. .036]	.022

	ΔCT	.215	.030*	[.179 3.320]	.032
	$LCC_{\text{drop}^{T2}}$ * ΔCT	-.221	.024*	[-3.726 -.270]	.057
**Truncated Time Series (10% Density Threshold)**	Age	-.244	.012*	[-.027 -.003]	.070
	Edu	-.013	.910	[-.048 .043]	.0001
	Sex	.094	.301	[-.077 .247]	.012
	NART	.307	.021*	[.003 .036]	.059
	$FLUID_{T1}$	-.599	<.001***	[-.474 -.212]	.235
	$Scrub\%_{T2}$	.068	.466	[-.008 .017]	.006
	$LCC_{\text{drop}^{T2}}$	-.041	.808	[-.169 .132]	.002
	$LCC_{\text{k=0}^{T2}}$	.020	.905	[-.017 .020]	.0002
	ΔCT	.175	.072	[-.132 2.986]	.025
	$LCC_{\text{drop}^{T2}}$ * ΔCT	-.257	.006**	[-3.687 -.633]	.082

ST1. List of predictors for linear regression models with ΔFLUID as the outcome variable. Treatment of the functional connectivity (FC) data is indicated in the green column. We report the results for (1) 5% density thresholding of the FC matrices; (2) 15% density thresholding of the FC matrices; and (3) when the 5-minute truncated time series with 10% density thresholding. Again, we controlled for scrubbing ($Scrub\%_{T2}$), initial LCC ($LCC_{\text{k=0}^{T2}}$), and baseline behavioral performance in each model. Change in cortical thickness (ΔCT: T2 − T1) was residualized with respect to baseline. Asterisks indicate statistical significance at threshold levels p<0.05 (*), p<0.01 (**), and p<0.001(***).

β= Standardized coefficient beta; *p*= p-value (uncorrected); *CI*= 95% confidence interval; $\eta_\rho{}^2$ = partial eta-squared effect size.
